# Towards a Pathogenic *Escherichia coli* Detection Platform Using Multiplex SYBR®Green Real-Time PCR Methods and High Resolution Melting Analysis

**DOI:** 10.1371/journal.pone.0039287

**Published:** 2012-06-25

**Authors:** Dafni-Maria Kagkli, Silvia Folloni, Elodie Barbau-Piednoir, Guy Van den Eede, Marc Van den Bulcke

**Affiliations:** 1 European Commission Joint Research Centre, Institute for Health and Consumer Protection, Molecular Biology and Genomics Unit, Ispra (VA), Italy; 2 Scientific Institute of Public Health, Brussels, Belgium; University of Hyderabad, India

## Abstract

*Escherichia coli* is a group of bacteria which has raised a lot of safety concerns in recent years. Five major intestinal pathogenic groups have been recognized amongst which the verocytotoxin or shiga-toxin (*stx*1 and/or *stx*2) producing *E. coli* (VTEC or STEC respectively) have received a lot of attention recently. Indeed, due to the high number of outbreaks related to VTEC strains, the European Food Safety Authority (EFSA) has requested the monitoring of the “top-five” serogroups (O26, O103, O111, O145 and O157) most often encountered in food borne diseases and addressed the need for validated VTEC detection methods. Here we report the development of a set of intercalating dye Real-time PCR methods capable of rapidly detecting the presence of the toxin genes together with intimin (*eae*) in the case of VTEC, or aggregative protein (*agg*R), in the case of the O104:H4 strain responsible for the outbreak in Germany in 2011. All reactions were optimized to perform at the same annealing temperature permitting the multiplex application in order to minimize the need of material and to allow for high-throughput analysis. In addition, High Resolution Melting (HRM) analysis allowing the discrimination among strains possessing similar virulence traits was established. The development, application to food samples and the flexibility in use of the methods are thoroughly discussed. Together, these Real-time PCR methods facilitate the detection of VTEC in a new highly efficient way and could represent the basis for developing a simple pathogenic *E. coli* platform.

## Introduction

*Escherichia coli* is a heterogeneous group of typically non-pathogenic gram-negative bacteria that are a natural part of the intestinal flora of animals and human [1]. Certain strains of the species have been recognized as human pathogens since the 1940s and have been linked to several food borne illnesses [2]. Five major groups of intestinal pathogenic *E. coli* have been recognized: enteropathogenic (EPEC), enteroaggregative (EAEC), enterotoxigenic (ETEC), enteroinvasive (EIEC), and verocytotoxin or shiga-toxin producing *E. coli* (VTEC or STEC respectively) [3]. Those VTEC that cause haemorrhagic colitis (HC) and hemolytic uremic syndrome (HUS) are called enteropathogenic *E. coli* (EHEC). During the last decade, the VTEC/EHEC group received special attention as some highly pathogenic serotypes caused severe epidemic outbreaks with numerous casual deaths [4], [5].

Verocytotoxin producing *E. coli* are zoonotic agents which were first recognized in the late 1970s by Konowalchuk and colleagues in Canada [6]. The sources of contamination include mainly cattle and food of bovine origin, with undercooked meat or ground beef being the major sources of human infections [7]. Other sources of contamination which have caused food borne infections are cider, lettuce, spinach, sprouts and recreational water [8].

The Shiga toxin family is composed of functionally related type I toxins of *Shigella dysenteriae* and closely related proteins produced by the EHEC. More than 200 serotypes produce shiga toxins, but only 50 have been associated with bloody diarrhoea or HUS in humans [9]. VTEC may contain two different genes, *stx*1 and *stx*2. Of both toxins, a number of allelic variants have been described, designated respectively as *stx*1a-1d and *stx*2 *stx*2a-2g [10], [11]. Although *stx*1 and *stx*2 possess a similar mode of action, they are immunologically distinct and are only 56% identical at the amino acid sequence level [12]. The toxins share a common polypeptide subunit structure consisting of an enzymatically-active A subunit (∼32 kDa) linked to five B subunits (∼7.5 kDa) [7], [13]. The B subunit is responsible for the binding to specific receptors in eucaryotic cell membranes, allowing the toxin to target different cell types; the A subunit, is proteolytically activated and the catalytically active A1 enzyme cleaves specifically the 28S RNA of the 60S ribosomal subunit, arresting protein synthesis what results in cell death [14], [15]. Despite the similarities of the two toxins, strains possessing the *stx*2 toxin have been more frequently associated to epidemiological diseases and are more likely to cause HUS, rather than strains producing *stx*1 and *stx*2 together or *stx*1 alone [12], [13], [15], [16], [17], [18].

Apart from the *stx* gene products, *E. coli* pathogenicity requires the colonization of the host intestinal mucosa through the formation of a characteristic histopathologic lesion, defined as “attaching and effacing” (A/E). Such colonization is accomplished in VTEC by the intimin, a protein encoded by the *eae* gene. Although the A/E lesion is not essential for bloody diarrhoea and HUS in humans, the majority of strains implicated in these syndromes, are *eae* positive [1]. Moreover, the simultaneous presence of *stx*2 and *eae* is considered a predictor of HUS, and the simultaneous presence of these two traits in a VTEC strain is more frequently correlated with virulence and HUS [19].

Due to the high number of outbreaks related to VTEC strains, the European Food Safety Authority (EFSA) has requested the monitoring of the “top-five” serogroups (O26, O103, O111, O145 and O157) most often encountered in foodborne diseases. EFSA also addressed the need for validated VTEC detection methods [20], [21]. In addition, the recent *E. coli* O104:H4 outbreak in the European Union during which 3774 cases were reported and fourty-four people died, highlighted the need for rapid, harmonized and validated methods which could facilitate rapid detection and characterization of food borne pathogens, especially in such emergency situations. Even though the recent *E. coli* O104:H4 strain is not a VTEC strain, it has been reported to possess characteristics of both the EHEC and EAEC group, and was thus also included in the present study together with the top-five previously mentioned serogroups.

A set of 8 TaqMan Real-time PCR recently validated VTEC methods [21], adopted by the European Normalization Committee as a technical specification (CEN TC275/WG6), was modified and further optimized for use with intercalating fluorescent dyes instead of multiple labelled probes. In the present study, the screening methods developed utilized SYBR®Green chemistry, and the reaction conditions were optimized for further characterisation of isolated VTEC/EHEC colonies by High Resolution Melting analysis (HRM), a refinement of the well-established melting curve analysis (T_m_ analysis). With HRM, small mutations in PCR products can be detected based on slight alterations in the profile of the dissociation curve of double strand DNA (dsDNA) molecules. To date, HRM analysis has been applied only in few cases in food microbiology, mainly aiming at the differentiation among species or strains [22], [23]. Nonetheless, HRM has been shown to be an extremely powerful tool in discriminating among very similar sequences [24], [25].

As the *stx* variants represent a set of highly homologous targets, an integrated approach capable of rapidly detecting the presence of the respective toxin genes and the adhesion/effacing/aggregating factors of VTEC or EAEC was developed. All HRM PCR reactions were optimized to perform under the same conditions, and several multiplex systems were developed to allow for more rapid analysis. The power of combining SYBR®Green screening with HRM analysis in discriminating among *E. coli* strains possessing different combination of traits is shown. Their application on food samples is demonstrated and the future expansion of the scope of this SYBR®Green/HRM screening approach is discussed.

## Results

The Real-time methods applied in this study have been previously shown to perform adequately in both clinical and food analyses using the Taqman™ technology [21], [26], [27], [28]. Here, the above-mentioned VTEC PCR methods were optimized for their combined application in SYBR®Green Real-time PCR either in a simplex or multiplex format. High Resolution Melting analysis was then pursued to further increase the discriminating power when applying these sets of VTEC detection methods.

### Simplex VTEC SYBR®Green Real-time PCR Method Development and Optimization

To verify the optimal primer concentrations for SYBR®Green Real-time PCR, all methods were tested at primer concentrations ranging from 150 nM to 1 mM. All amplifications using an appropriate positive control as DNA template were assessed for generation of a single amplicon with a specific melting temperature and the expected size on gel. All methods tended to induce the formation of primer dimers when high primer concentrations were used. Therefore, 150 nM was chosen as the most appropriate concentration for use, since no primer dimers were formed and good amplification was obtained. In this optimized set-up, all methods operate under identical PCR conditions allowing combination of all targets within a single analytical run.

### Multiplex SYBR®Green Real-time PCR Method Development and Optimization

Two types of SYBR®Green Real-time multiplex reactions were developed. The first one was a duplex *stx1/stx2*/*eae* method with primer concentrations 500 nM for the *stx* and 150 nM for the *eae* target respectively. Such duplex analysis enables the immediate detection of the presence of *E. coli* strains containing one or both VTEC virulent targets. Addressing the emergency of the pathogenic *E. coli* O104:H4 strain in Germany, a triplex method containing the above mentioned VTEC virulent traits together with the aggregation substance target (*agg*R) present in the O104 strain was developed. Optimal primer concentrations for this four target combination were 500 nM for of the *stx 1&2* primers, 250 nM for the *eae* and 150 nM for the *agg*R target.

### Assessment of the Melting Temperature as VTEC Virulent Target Identification

To evaluate the correctness of the PCR amplicons obtained with the respective methods, the melting temperature (T_m_) of each target was determined experimentally. Therefore, for the “top five” VTEC strains, single target plasmids harbouring the expected amplicons were synthesized and applied as reference template DNA for each amplicon (see Table 1). Results were compared with those obtained from genomic DNA from reference strains (Table 2). In the case of the *agg*R target, only genomic DNA of the strain causing the German outbreak was used. Discrete peaks with a specific T_m_ value for each target were obtained with every different method. All experiments were performed in triplicate and on three different thermal cyclers: Applied Biosystems AB7500, Biorad iQ5, and Roche LightCycler480. However, T_m_ values obtained for each method and at each platform were significantly different amongst them (*p<0.05*), indicating a different mathematical curve-fitting and integration of the fluorescence data. Nonetheless, within the same platform, no significantly different Tm values were obtained when control samples and unknown samples were tested (*p>0.05*).

**Table 1:** Melting curve analysis of VTEC target plasmids and pure cultures of reference strains using the SYBR®Green Real-time simplex or multiplex reactions on an AB 7500, Biorad iQ5 and Roche LightCycler 480 PCR thermal cycler (STP: standard plasmid).

AB7500				Roche LC480			Biorad iQ5		
DNA	*stx* or *eae*	*stx*/*eae*	*stx*/*eae*/*agg*R	*stx* or *eae*	*stx*/*eae*	*stx*/*eae*/*agg*R	*stx or eae*	*stx/eae*	*stx/eae/aggR*
**STP (** ***stx*** **1)**	77.3±0	N/A	N/A	80.65±0.057	N/A	N/A	81.67±0.289	N/A	N/A
***STP (stx2)***	78.72±0.606	N/A	N/A	83.14±0.063	N/A	N/A	83.50±0.001	N/A	N/A
***STP (eae)***	75.70±0.001	N/A	N/A	80.11±0.02	N/A	N/A	79.50±0.001	N/A	N/A
***Genomic DNA***	77.15±0.446or75.63±0.265	76.08±0.809	78.53±0.953	82.28±0.760or79.30±0.078	82.22±0.05078.24±0.010	81.23±0.05979.59±0.03582.88±0.355	81.33±0.258 or 80.00±0.001	81.70±0.274 or 79.5±0.289	81.58±0.20479.83±0.25883.00±0.001

**Table 2:** Strains used in the study.

Strain and serogroup	Coding	Source of genomic DNA	Virulence traits
*Escherichia coli*	ATCC 25922	Food Pathogen Laboratory (Belgium) 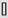	
*E. coli* O157:H7	EH 630	Food Pathogen Laboratory (Belgium) 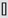	*eae*
*E. coli* O145:H-	EH 1492: TIAC 612	Food Pathogen Laboratory (Belgium) 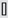	*eae, stx1*
*E. coli* O145:H28	EH1533: TIAC 613	Food Pathogen Laboratory (Belgium) 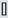	*eae, stx2*
*E. coli* O103:Hnt	EH1717: TIAC 614	Food Pathogen Laboratory (Belgium) 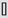	*eae, stx1*
*E. coli* O111:Hnt	EH1811: TIAC 615	Food Pathogen Laboratory (Belgium) 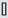	*eae, stx1, stx2*
*E. coli* O26:H	EH1815: TIAC 616	Food Pathogen Laboratory (Belgium) 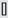	*eae, stx1*
*E. coli* O157:H7	EH1819: TIAC 617	Food Pathogen Laboratory (Belgium) 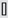	*eae, stx2*
*E. coli* O26:H	EH1823: TIAC 618	Food Pathogen Laboratory (Belgium) 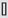	*eae, stx1*
*E. coli* O157:H-	EH1829: TIAC 619	Food Pathogen Laboratory (Belgium) 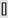	*eae, stx1, stx2*
*E. coli* O103:H	EH1831: TIAC 620	Food Pathogen Laboratory (Belgium) 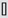	*eae, stx1*
*E. coli* O026:H	EH1839: TIAC 621	Food Pathogen Laboratory (Belgium) 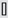	*eae, stx1*
*E. coli* O157:H7	EH1844: TIAC 622	Food Pathogen Laboratory (Belgium) 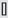	*eae, stx2*
*E. coli* O145:H-	EH1846: TIAC 623	Food Pathogen Laboratory (Belgium) 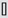	*eae, stx2*
*E. coli* O111:H	EH1847: TIAC 624	Food Pathogen Laboratory (Belgium) 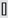	*eae, stx1*
*E. coli* O111:H-	EH1760: TIAC 625	Food Pathogen Laboratory (Belgium) 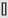	*eae, stx1*
*E. coli* O103:Hnt	EH1783: TIAC 626	Food Pathogen Laboratory (Belgium) 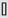	*eae, stx1*
***E. coli*** ** O157**	**C210-03**	EURL *E. coli*	***eae, stx1b, stx2c***
*E. coli* O157	ED 620	EURL *E. coli*	*eae*
*E. coli* O157	ED 621	EURL *E. coli*	*eae*
***E. coli*** ** O145**	**C1178-04**	EURL *E. coli*	***stx1a, eae***
*E. coli* O145	ED 645	EURL *E. coli*	*stx2a, eae*
*E. coli* O145	ED 657	EURL *E. coli*	*stx2a, eae*
***E. coli*** ** O103**	**C125-06**	EURL *E. coli*	***stx2, eae***
*E. coli* O103	ED 287	EURL *E. coli*	*stx1a, eae*
*E. coli* O103	ED259	EURL *E. coli*	*stx1a, eae*
***E. coli*** ** O111**	**MM13-02**	EURL *E. coli*	***eae***
*E. coli* O111	ED 476	EURL *E. coli*	*stx1a, stx2a, eae*
***E. coli*** ** O26**	**C1188-02**	EURL *E. coli*	***stx1a, stx2a, eae***
*E. coli* O26	ED-643	EURL *E. coli*	*stx1a, eae*
*E. coli* O26	ED 654	EURL *E. coli*	*stx2a, eae*
*E. coli O104:H4*	11 2027	EURL *E. coli*	*stx2, agg*R
*E. coli O104:H2*		Food Pathogen Laboratory (Belgium) 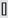	
*Escherichia hermanii*	TIAC 668: 08/0097	Food Pathogen Laboratory (Belgium) 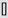	
*E. hermanii*	TIAC 669: 03/065	Food Pathogen Laboratory (Belgium) 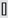	
*E. hermanii*	TIAC 671: Div 2663	Food Pathogen Laboratory (Belgium) 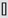	
*Escherichia fergusonii*	TIAC 673: 95/394	Food Pathogen Laboratory (Belgium) 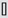	
*E. fergusonii*	TIAC 674: Div 3541	Food Pathogen Laboratory (Belgium) 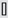	
*Escherichia vulneris*	TIAC 675: Seq048	Food Pathogen Laboratory (Belgium) 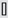	
*E. hermanii*	TIAC 670: EH 148	Food Pathogen Laboratory (Belgium) 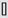	
*E. fergusonii*	TIAC 672: 05/1161	Food Pathogen Laboratory (Belgium) 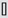	
*Shigella sonnei*	10-03865	Food Pathogen Laboratory (Belgium) 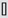	
*Shigella flexneri*	10-03891	Food Pathogen Laboratory (Belgium) 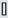	
*Shigella boydii*	10-02874	Food Pathogen Laboratory (Belgium) 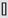	
*Shigella dysenteriae*	10-01857	Food Pathogen Laboratory (Belgium) 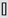	
*Hafnia alvei*	IZS 13	Istituto Zooprofilattico Sperimentale Lombardia	
*Enterobacter sakazakii*	CIP 103183	Institut Pasteur	
*Listeria monocytogenes*	ATCC 19115	Istituto Zooprofilattico Sperimentale Lombardia	
*Salmonella enterica* subsp. *enterica*	ATCC6994	Istituto Zooprofilattico Sperimentale Lombardia	
*Salmonella seftenberg*	ATCC 43845	Istituto Zooprofilattico Sperimentale Lombardia	
*Salmonella hadar*	IZS	Istituto Zooprofilattico Sperimentale Lombardia	
*Salmonella enteritidis*	IZS 581	Istituto Zooprofilattico Sperimentale Lombardia	
*Salmonella cerro*	IZS 1138	Istituto Zooprofilattico Sperimentale Lombardia	
*S. boydii*	BAA-1247	Istituto Zooprofilattico Sperimentale Lombardia	
*S. dysenteriae*	ATCC 13313	Istituto Zooprofilattico Sperimentale Lombardia	
*Klebsiella pneumoniae*	ATCC 10031	Istituto Zooprofilattico Sperimentale Lombardia	
*Citrobacter freundii*	ATCC 43864	Istituto Zooprofilattico Sperimentale Lombardia	
*Yersinia enterocolitica*	ATCC 9610	Istituto Zooprofilattico Sperimentale Lombardia	
*Proteus mirabilis*	ATCC 7002	Istituto Zooprofilattico Sperimentale Lombardia	
*Campylobacter jejuni*	ATCC 49943	Istituto Zooprofilattico Sperimentale Lombardia	
*Staphylococcus aureus*	ATCC 25923	Istituto Zooprofilattico Sperimentale Lombardia	

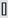
 Belgian NRL from VTEC, *Campylobacter*, *Salmonella* and *Listeria* from food.

### Specificity and Sensitivity of the Simplex SYBR®Green Real-time PCR Methods

Specificity of all methods was assessed by testing the reactions with all the strains presented in Table 2. No false positive or false negative results were observed; the T_m_ values of the respective targets were in accordance with the values obtained with the plasmid and the reference genomic DNA. In the cases when both *stx*1 and *stx*2 are present in the same strain, a single broader peak was obtained, similar to the reference strains with corresponding T_m_ values. Also, a detailed Bioinformatics analysis performed with the respective primers did not reveal any similarities with non-target DNA sequences (NBCI Database access in 11/2010).

**Figure 1:**
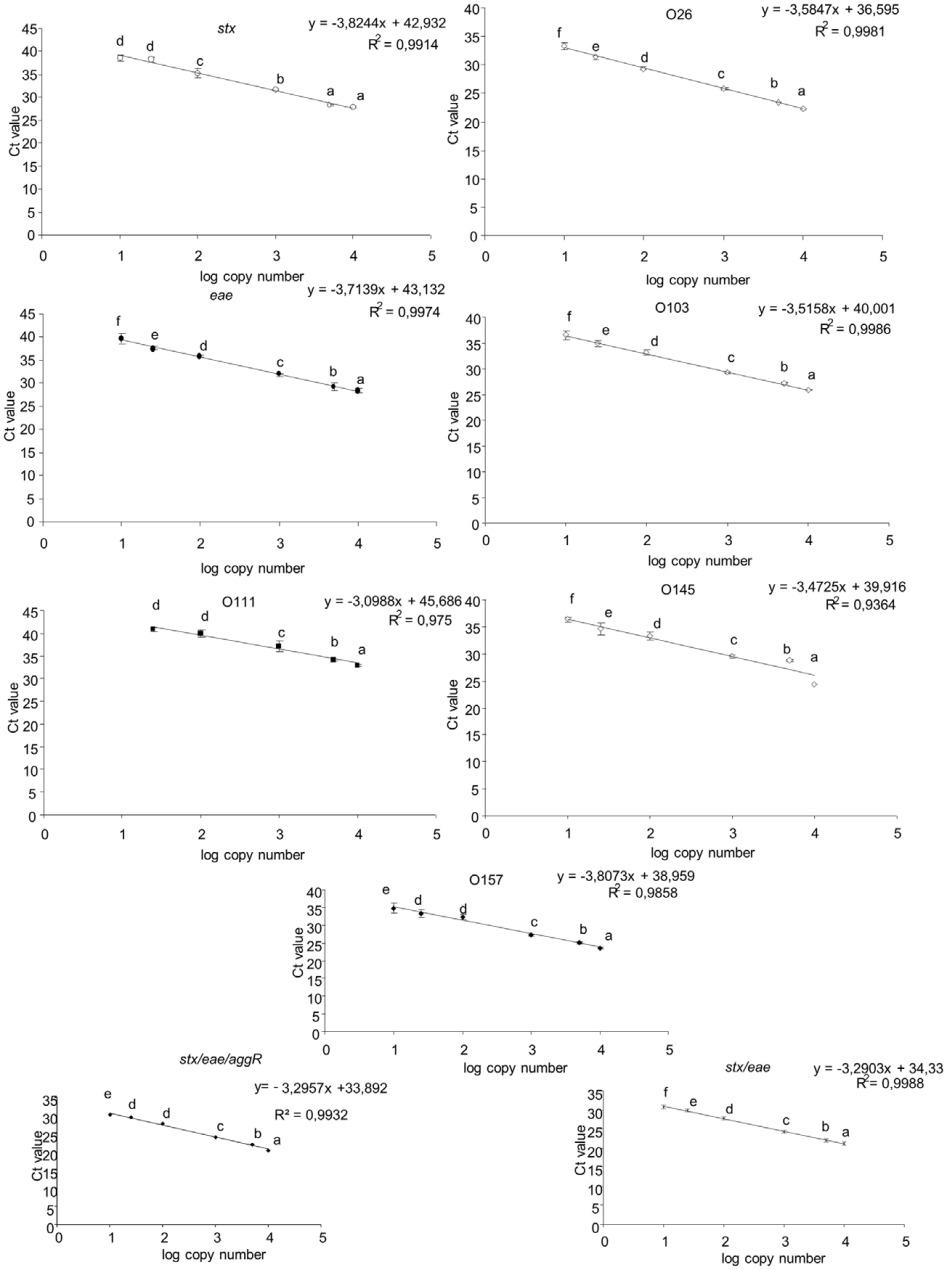
Dynamic range of all methods. Letters indicate significantly different values (p<0.05).

To assess their sensitivity, the dynamic range and the PCR efficiency of the respective simplex Real-time PCR methods were assessed based on six replicate analyses of a dilution series ranging from 10000 down to 10 copies [29]. Six out of six positive signals were always obtained down to 10 copies per reaction except for the O111 PCR serotyping method for which positive signals were obtained only down to 25 copies (3 out of 6 replicates). The statistical analysis of the Ct values indicated that each copy number tested was significantly different (*p<0.05*) from its successive one for methods O26, O103, O145, *eae* and *stx*1/*stx*2/*eae* (Figure 1); for the *stx* and O111 methods, the last point of the dynamic range was not statistically different from its previous one, whereas for methods *stx*1/*stx*2/*ea*e/*agg*R and O157, the 100 copies were not statistically different from the 25 copies tested (*p>0.05*). In the latter cases though, the last copy number tested was significantly different from its previous one. The calculated PCR efficiencies ranged between 83% and 110% (Figure 1). The highest PCR efficiency was obtained with the O111 serogroup specific method; the lowest efficiency was measured for the *stx* method, most probably due to the use of degenerated primers in this particular method allowing for the simultaneous detection of both the *stx1 & 2* toxins in a single reaction (Figure 1). All VTEC detection methods performed equally well either in SYBR®Green or Taqman™ Real-time PCR [this study], [21].

### Specificity and Sensitivity of the Multiplex SYBR®Green Real-time PCR Methods

**Figure 2:**
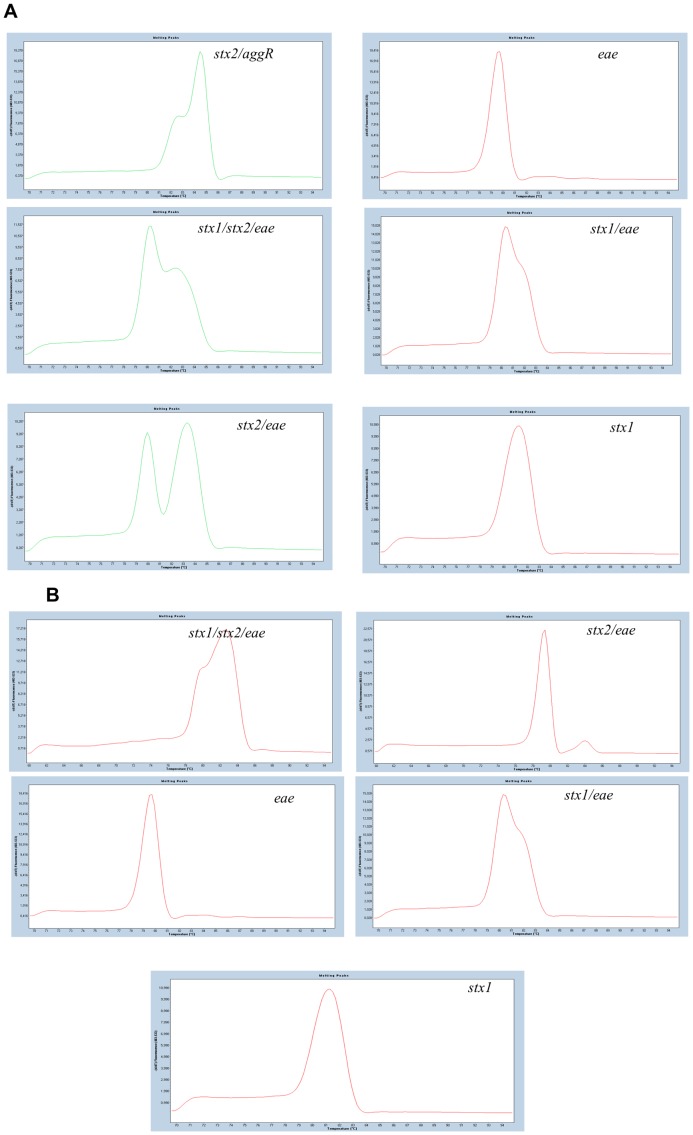
Melting peaks of amplicons in (A) triplex and (B) duplex PCR. Respective targets are indicated on each graph.

Two multiplex systems were developed: a duplex system targeting stx/eae and a triplex system including in addition the *agg*R target. With the multiplex PCR different melting curve profiles were obtained depending on the nature and the number of the different targets present in the test strain (Figure 2). The profile and the observed T_m_ values varied depending on the type of the *stx* toxin(s) present in the strain. It has to be noted though, that only the LightCycler480 managed to give distinct T_m_ values, as the AB7500 and iQ5 Biorad analysis software recognized in most cases only the highest peak detected. Whenever a strain possessed *stx*1 and *eae* together, a single large peak with a T_m_ = 80.92±0.071 was obtained. When *stx*2 and *eae* targets were simultaneously present, two distinct peaks were obtained; firstly the *eae* peak with a T_m_ = 79.30±0.078 and secondly the *stx2* peak with a T_m_ = 83.12±0.014. Whenever *stx*1, *stx*2 and *eae* were all present, a single large peak with a T_m_ = 81.87±0.087 was obtained (for a detailed representation of the generated profiles see Figure 2). The triplex PCR was tested on the *E. coli* O104:H4 strain which was reported to contain the *stx*2 toxin and the *agg*R but lacking the *eae* target. The melting analysis gave one large peak at T_m_ = 82.88±0.355 and a “shoulder-like” lower one, with T_m_ = 81.23±0.059. No interference of amplification of the different targets was observed in the multiplex formats; positive amplification of all targets was obtained where expected, with no false positives or false negatives.

The dynamic range for the multiplex reactions was estimated using the same serial dilution approach as applied in the case of the simplex methods. It was shown that for both multiplexes a linear correlation over the tested range was obtained (Figure 1). It should be noted that in multiplex reactions the measured C_t_-values represent the combined signals of all targets and no conclusion on the efficiency of amplification of the individual targets can be deduced.

### Applicability of the VTEC SYBR®Green Real-time PCR Methods on DNA Extracted from Artificially Inoculated Food Matrices

Five ng of extracted DNA from 1 ml of enriched, artificially inoculated matrices were used to test the applicability of these methods in food matrices with three different platforms (Table 3). All methods correctly identified the expected targets since the T_m_ values were identical to the ones of the corresponding controls. The non-inoculated negative control samples of the salad and milk matrices were not positive for any VTEC targets. In the meat matrix, the *stx* and *eae* virulence targets and the O26 and O103 serogroup targets were detected but at very low C_t_ values. For the respective samples, the C_t_ difference between inoculated and non-inoculated sample was in all cases higher than 8. No growth was observed on the selective plates however, indicating that the PCR signals in the case of the meat samples resulted from DNA of non-viable material.

### High Resolution Melting Curve Analysis of VTEC and EAEC Targets

**Figure 3:**
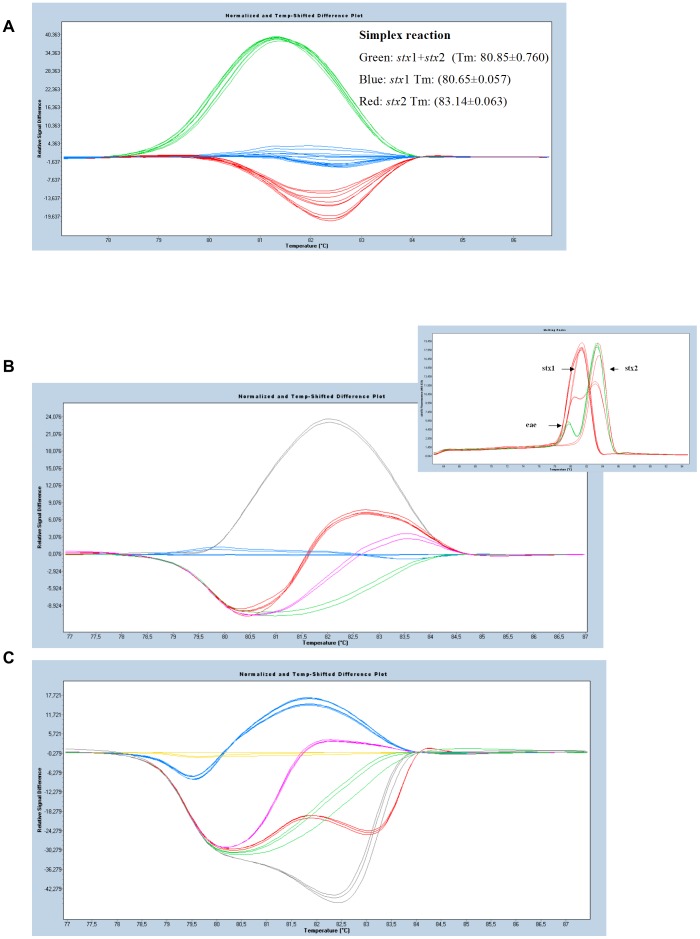
HRM of reference strains: (A) Simplex reactions, (B) Duplex reactions. Grey group: *stx*1+*stx*2+*eae*, blue: *stx*2+*eae*, red: *stx*1+*eae*, green: *stx*2, pink:*stx*1, (C) Triplex reations. Blue: *stx*1+*stx*2+*eae,* yellow: stx2*+eae,* pink*: stx1+eae,* green*: stx1, red: stx2+agg*R, grey*: eae.*

Isolates possessing the virulent traits *stx*1 and/or *stx*2, *eae* and *agg*R were further characterized by high resolution melting (HRM) analysis of the amplification profiles obtained after simplex or multiplex PCR. Indeed, whenever *stx*1 and *stx*2 were simultaneously present, the classical melting analysis of the simplex reactions based only on the T_m_ value could not distinguish between both types of toxins. Therefore, an additional post-PCR analysis, the so-called HRM was performed which enabled the correct grouping of the isolates according to the different combinations of toxins present in the respective strains (Figure 3A). In the multiplex reactions the respective strains were further classified into distinct groups depending on 1) which toxin was present (*stx*1 and/or *stx*2) and 2) whether or not they also possessed an intimin (*eae*) or an aggregating substance target (*agg*R). After HRM analysis, *E. coli* strains possessing *stx*1 and *eae* could easily be distinguished from the ones possessing *stx*1, *stx*2 and *eae* together (Figure 3B), what was not possible by classical melting curve analysis (Figure 2B).

In all cases, this differentiation originates from the capacity of HRM to distinguish the *stx* sequences even when only few base pair differences are present. HRM analysis also clearly distinguished the *agg*R*(+)/eae(-) E. coli* O104 strain from *agg*R*(-)/eae(+)* strains (Figure 3C). All HRM results obtained were verified using Agilent Bioanalyzer 2100 confirming the presence and the size of all targets (data not shown).

## Discussion

VTEC *E. coli* are food borne pathogens which cause sporadic infections and severe outbreaks in humans [1], [13]. The main serogroups involved are O26, O103, O111, O145, and O157 [30]. Nonetheless, the recent crisis in Germany (Spring 2011) brought to light one more serogroup, the O104 strain, which had to-date not been associated with outbreaks similar to the ones caused by the above mentioned serogroups. The O104 strain is not a typical VTEC strain: it has acquired traits from the VTEC, the *stx* toxin, does not have an *eae* gene but instead harboured the *agg*R gene from the EAEC. Published data on O104 serogroup are still scarce since it was not a frequently isolated serogroup [31], [32], [33], [34], [35].

**Table 3:** Results of the artificially inoculated matrices.

Method/Platform	Milk			Meat			Salad		
	AB7500	iQ5	LC480	AB7500	iQ5	LC480	AB7500	iQ5	LC480
*stx*	77.48±0.210	81.50±0.001	80.82±0.102	78.22±0.776	81.50±0.001	80.80±0.045	77.45±0.766	81.50±0.001	80.79±0.053
*eae*	75.24±0.213	80.00±0.001	79.84±0.001	75.13±0.279	80.00±0.001	79.78±0.017	75.40±0.360	79.70±0.288	79.81±0.010
*O26*	73.16±0.206	78.17±0.288	77.23±0.050	73.85±0.122	78.50±0.001	77.37±0.045	73.47±0.231	78.50±0.001	77.26±0.044
*O103*	72.30±0.300	77.33±0.288	76.10±0.017	73.03±0.288	77.50±0.001	76,33±0,047	72.53±0.351	77.50±0.001	76.17±0,156
*O111*	73.92±0.533	78.50±0.001	77.60±0.040	74.60±0	79.00±0.001	78.58±0.087	74.17±0.231	78.83±0.289	78.55±0.090
*O145*	81.55±0.210	85.67±0.288	86.21±0.061	82.60±0	86.00±0.001	86.82±0,098	81.90±0.400	85.83±0.289	86.86±0.055
*O157*	74.60±0	78.00±0.001	78.18±0.115	74.60±0	78.50±0.001	78.41±0.029	74.17±0.23	78.00±0.001	78.39±0.100
*stx/eae*	78.3±0.173	81.50±0.001	80.04±0.294 and 83.48±0.274	79.10±0	81.50±0.001	80.25±0.030 and 83.69±0.021	78.3±0.173	81.50±0.001	80.11±0.010 and 83.55±0.030
*stx/eae/agg*	NA	NA	NA	NA	NA	NA	NA	NA	NA

Analyses were performed on an AB7500, iQ5 and LC480. On the left column, the method for which all matrices were screened. Tm values presented are means of the Tm values of three replicates ± standard deviation.

The present study focused on the development of Real-time PCR methods which could be used either in simplex or multiplex formats under the same PCR conditions to detect a) the most widely distributed virulence traits possessed by the strains under investigation: *stx*, *eae*, *aggR*, and b) the serogroup they belong to, focusing on the five major ones mentioned above. In addition, by coupling Real-time PCR to post-PCR HRM analysis, the strains used in this study could be correctly grouped according to the respective virulence traits they possessed.

For detection purposes, they can be used to analyse food samples after enrichment and provide an indication on the presence or absence of pathogenic *E. coli* in the sample. If the outcome is negative, no further investigation is required. In the case of positive outcomes, confirmation on single colonies is absolutely necessary to determine viability and presence of the virulence traits in the same cell, as previously discussed [21]. Serogroup determination can then be performed on the isolated single colonies.

A number of VTEC Real-time PCR SYBR® Green methods have already been developed based on the detection of the toxins and the intimin [36], [37], [38]; these methods exploit the melting analysis only to assess the presence of the virulent traits. The novelty of the current study lies in the fact that the post- PCR analysis by HRM allows not only the detection but also the correct sub-grouping of the strains based on the toxin variant they possess.

HRM analysis, which is used in clinical and cancer research, has however been scarcely applied in food microbiology, and one of the few applications is “Multilocus Sequence Typing” to discriminate amongst *Campylobacter jejuni* strains causing gastroenteritis in developed countries [24]. Here, HRM allowed the successful distinction of pathogenic strains containing *stx*1 alone or together with *stx*2 targets. These differences are only detectable by HRM analysis, since the T_m_ values of both toxins in classical melting analysis are very similar. Such information may be of interest as several EHEC strains have been reported to be more pathogenic and more frequently related to severe diseases when they possess the *stx*2 instead of the *stx*1 toxin [12], [16]. Therefore, the application of HRM analysis allows decision makers to proceed faster depending on which toxin or combination of toxins a strain contains. A rapid response may be especially critical in epidemiological and emergency situations [32], [34]. HRM was furthermore demonstrated to be a valuable tool in sub-grouping strains, allowing cost-efficient management of any subsequent analyses such as DNA sequencing. Nevertheless, to apply HRM, reference strains need always to be included in the analysis. Clustering into groups based on the presence of particular targets (in our study, the possession of virulent traits) is only feasible when profiles obtained from an unknown strain can be matched to those generated with well-characterized reference strains.

High Resolution melting analysis is very sensitive and special attention should be paid to a number of factors affecting the PCR setup. Firstly, PCR reactions need to be optimized for the MgCl_2_ concentration in order to obtain the most efficient and stringent PCR conditions. Next, the template DNA should be of good quality and must be quantified precisely beforehand. Regarding the quality of the DNA, samples and reference DNA should preferably have been extracted using the same procedure; in this way, the composition of the compared DNA samples with respect to any interfering compounds will be most alike (data not shown). Quantification of the extracted DNA is important to guarantee that equal numbers of template are used in the different PCR reactions. Then, with PCR reactions having similar efficiencies, the fluorescent level reached at the end point will be most similar, resulting in optimal HRM analysis [25]. In our study, we tested different concentrations of DNA deriving from pure cultures, and we concluded that at least 2000 copies of initial target DNA, when we refer to pure culture, are necessary in order to obtain reliable results (data not shown).

Next, the respective VTEC virulence traits methods were optimized for application in multiplex formats. Both a duplex (*stx*/*eae*) and a triplex (*stx*/*eae*/*agg*R) system were successfully established. In this way, quick responses can be provided even when little sample material and/or time is available. This may be extremely important when many rapid, reliable analyses are needed, e.g., as was the case in the recent *E. coli* crisis [31], [34]. For this, the VTEC system was expanded to a triplex (stx/eae/*agg*R) system covering in addition to the previously top-five serogroups also the “atypical” O104 strain.

In the case of the multiplex reactions, optimization of the methods was required to allow for optimal HRM profiling. In particular, when multiplexing, it is crucial to combine reactions with similar efficiencies, since the amplification would be the result of all targets present in the template DNA. In our system the nature of the amplification products can be verified in the preceding melting step by a classical T_m_ analysis. It remains however critical to include the appropriate positive controls for each of the target sequences under analysis.

The methods optimized here, previously developed for clinical samples [26], have been tested in three different types of food samples on DNA extracted from single colonies as well as on DNA extracted from the enriched matrices. We used three different PCR platforms and all of them performed equally well, since all targets were correctly identified. No statistically significant Tm values were obtained between the control DNA of the pure culture or the plasmid, and the DNA extracted from the inoculated matrices (*p>0.05*). Even though detection of the pathogen and correct amplification was successful in the enriched samples, we do not recommend the use of HRM analysis on such complex samples. Indeed the presence of background microflora especially when non-viable organisms with similar targets are present, will influence the generated HRM profiles and any sub-grouping can be inconclusive.

Thus, the intercalating dye based methods developed in this study broaden the application of Real-time PCR methods in the detection of pathogenic *E. coli* compared to commonly used probe-based methods. In fact, a comparison of the results of the same methods developed for Taqman-based probes [21] indicates clearly that the specificity and sensitivity of the methods are comparable; No false positive or false negative results were obtained in any case and the dynamic ranges were similar. Moreover, in the case of multiplexing where more probes are necessary, and complex quenchers are to be preferred, the cost of the analysis is higher than that of the intercalating dye based methods. In addition, they perform equally well and can be combined with the HRM analysis, allowing grouping of the isolates based on their virulence traits. In our opinion, HRM analysis is thus a technology which could have broader applicability in microbial detection in general. However, to allow the use of HRM in routine applications for pathogen detection, a better understanding of the algorithms of the HRM software is required. Finally, the approach presented here could easily be extended by adding virulence traits of other groups of *E. coli* causing outbreaks, enabling the rapid detection and clustering of *E. coli* strains of epidemiological interest.

## Materials and Methods

### Bacterial Strains and Growth Conditions

*E. coli* strains of human or bovine origin, belonging to O26, O103, O111, O145, O157 and O104 serogroups together with closely related species were included in the study (Table 2). Pure overnight cultures of the non-*E. coli* bacterial strains to be used in the specificity testing were grown in Tryptone soy broth (TSB) at 37°C. *E. coli* strains were grown in LB broth.

### DNA Extraction and Quantification

DNA extractions were performed using Pure™ Complete DNA and RNA Purification Kit (Epicentre, Madison, WI, USA). All extractions were performed in duplicate and the DNA was quantified with PicoGreen (Invitrogen, Italy). All DNA extracts were diluted to a stock concentration of 20 ng/µl in Molecular Biology Grade water and stored at −20°C. Serial dilutions were performed as previously described [21]. Plasmids previously described were used as positive control for each target [21].

### SYBR®Green Real-time PCR Conditions

Primers of the respective targets were taken from [26], [27], [28], [39]. They were purchased from Microsynth (Microsynth AG, Balgach, Switzerland). Development and optimization of the Real-time PCR reactions were achieved using 7500 Real-time PCR System (Applied Biosystems). All PCR amplifications were performed in a final volume of 25 µl containing 1X of Power SYBR®Green PCR Master Mix (Applied Biosystems, Milan, Italy), and the appropriate concentration of each primer and quantity of DNA template. For simplex reactions, 150 nM of each primer were used; in case of the *stx*/*eae* multiplex reaction, 150 nM for the *eae* primers and 500 nM for the *stx* primers were used. For the *stx*/*agg*R/*eae* multiplex reactions, the primers were used at 500 nM/100 nM/250 nM, respectively. All Real-time PCR reactions were performed under the following conditions: 95^o^C for 10 min, and then 45 consecutive cycles of first 15 sec at 95^o^C and then 1 min at 60^o^C. Melting curve analysis was done using the default settings of the device (7500 System SDS Software v. 1.3, Applied Biosystems).

After setting-up, the method was subsequently transferred to two more platform, iQ5 of Biorad and LightCycler480 of Roche, and all analyses were repeated here to test the robustness of the system to different analysis softwares.

### Verification of the Sensitivity of the Respective SYBR®Green PCR Assays

To test the sensitivity of the Real-time PCR methods in terms of (approximate) target copy number, serial dilutions of genomic DNA of well-characterized positive controls were analyzed. The limit of detection (LOD) was set at the required target copy number per PCR reaction well to obtain a reproducible ( = six-times repeatable) specific amplification (see below). From these analyses, also the PCR efficiency (E) for each of the methods was calculated according to:
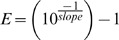
The PCR efficiency (E) could then be expressed in percentage:



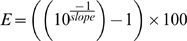
The dynamic range over which the target could be detected and the efficiencies of the respective PCR reactions were calculated using a 5-log dilution series starting from 10^5^ down to an estimated single copy.

### Determination of the Specificity of the Respective SYBR®Green PCR Assays

Specificity of the primers and the amplicons of interest was initially performed by BLAST analysis using the NCBI software package. Plasmids harbouring the corresponding amplicons for the VTEC PCR methods were synthesized at GeneArt as previously described [21]. Primer pair specificity for each target was assessed by testing amplification of genomic DNA of target-containing and target-lacking strains (Table 2). The following criteria were set to define what is considered as a “specific signal” generated in SYBR®Green Real-time PCR analysis: (1) an (exponential) amplification above the threshold level obtained with template DNA comprising the target sequence(s), (2) a lack of amplification with the non-template control (NTC) and the genomic DNA from strains reported to lack that particular target (Table 2), (3) with any target-containing template DNA, the amplified PCR product represents a single peak upon melting analysis with a unique melting temperature (T_m_) value corresponding to the nominal T_m_-value obtained with the corresponding plasmid as template DNA (with an acceptable standard deviation (SD ±1°C), (4) a single band on agarose gels after PCR amplification using single target-containing template DNA, two and three for the *stx/eae* and *stx/eae/agg*R reactions respectively with (5) a molecular weight corresponding to the predicted size for each PCR amplicon. All specificity tests were performed in triplicate using about 10^4^ copies of genomic DNA as template.

### Detection in Artificially Inoculated Food Matrices, DNA Extraction and Quantification

Overnight *E. coli* cultures of the different serogroups were inoculated in LB broth and incubated at 37^o^C. Twenty five grams or ml of three different types of matrices (minced meat, ready-to eat salad, and pasteurized skimmed milk) were artificially inoculated each with an *E. coli* strain belonging to a different serogroup as previously described [21]. One sample from each matrix was not inoculated but treated similarly to the inoculated ones, and represented the negative control sample. Samples were incubated at 37^o^C for 18–24 h, as described previously [21], before twice 1 ml was taken from each sample and DNA extractions were performed using Pure™ Complete DNA and RNA Purification Kit (Epicentre, Madison, WI, USA). All extractions were performed in duplicate. The concentration of the extracted DNA was quantified fluorimetrically using the PicoGreen dye (Invitrogen, Italy). Matrices were tested for presence/absence of VTEC before artificial inoculation. Total bacterial load of each matrix was also estimated on Plate count agar (Oxoid). Confirmation of presence/absence of the inoculated VTEC strain was obtained by streaking from the enrichment broth on selective TBX agar plates (Oxoid). Real time PCRs were performed to assess the detection of the pathogens in food matrices.

### High Resolution Melting (HRM) Analysis

HRM PCR analyses were performed in a 96-well plate using the LightCycler® 480 Real-time PCR System (Roche Diagnosctics, Germany). The final reaction volume was 20 µl containing 1× LightCycler® 480 High Resolution Melting Master, 4 mM of MgCl_2_, 0.2 ng of DNA, and the appropriate primer concentration depending on the type of method used as described above. In this case, the PCR program consisted of an initial denaturation of 10 min at 95°C, followed by 30 cycles of 10 s at 95°C, 10 s at 60°C and 15 s at 72°C. Prior to the melting analysis, heteroduplex formation was enhanced by heating the amplification products at 95°C for 1 min and cooling down to 40°C for 1 min. For the HRM analysis, the plate was heated from 60°C to 95°C allowing 25 data acquisitions per 1°C. HRM data analysis was performed using the LightCycler® 480 Gene Scanning software version 1.2 (Roche Diagnostics, Germany). Any samples with low initial target copies (set at a C_t_ ≥25 as monitored by Real-time PCR) were not used in HRM analyses. All HRM data analyses were performed applying the default parameters recommended by the manufacturer. All analyses were done in triplicate; positive/negative controls and non-template controls (NTC) were included in each run.

### Statistical Analysis

All data generated by the three different platforms tested were statistically analysed using Microsoft Excel 2010. A comparison of the Ct values was performed and a multiple comparison was done to determine which values were significantly different from which others (p<0.05). Fisher’s least significant difference (LSD) procedure was used to discriminate among the values and to assign significantly different values.
